# Chicken Primordial Germ Cell Surface Marker

**DOI:** 10.3390/ani15131868

**Published:** 2025-06-24

**Authors:** Tamara J. Gough, Terry G. Wise, Matthew P. Bruce, Timothy J. Doran, Daniel S. Layton, Andrew G. D. Bean

**Affiliations:** Health and Biosecurity, Australian Centre for Disease Preparedness, Commonwealth Scientific and Industrial Research Organisation, Geelong, VIC 3220, Australiaandrew.bean@csiro.au (A.G.D.B.)

**Keywords:** primordial germ cell, chicken vasa homologue, chicken myosin heavy chain 9, surface marker, avian biotechnology, germ cell sorting

## Abstract

The creation of transgenic chickens holds significant promise for the agricultural and biotechnological sectors, offering potential improvements in disease resistance and production efficiency. The preferred method for generating gene-edited chickens involves the genetic manipulation of primordial germ cells (PGCs), making the identification and isolation of these cells a growing focus of research. PGCs are the precursors to sperm and oocytes, responsible for transmitting genetic material to the next generation. In humans, PGCs are characterized by their large size, round nuclei, and refractive lipids in the cytoplasm, and can be identified using periodic acid–Schiff (PAS) staining and the surface marker stage-specific embryonic antigen 1 (SSEA1). Similarly, chicken PGCs express SSEA1, but their most specific marker is the chicken vasa homologue (CVH), the avian equivalent of the RNA-binding factor gene *vasa*. However, SSEA1, along with other known surface markers, does not bind to all PGCs or lacks specificity, while CVH, although highly specific to PGCs, is intracellular and unsuitable for isolating viable cells. This study aims to develop an antibody targeting a PGC surface marker with the same specificity as CVH. Despite the importance of identifying surface markers for PGC characterization, to date, such reagents are limited. To address this, whole chicken PGCs were injected into mice, leading to the generation of a panel of monoclonal antibodies. One antibody was found to bind cultured chicken PGCs and showed reduced expression upon differentiation with retinoic acid, indicating its specificity to PGCs. Immunoprecipitation followed by mass spectrometry identified the antigen as myosin heavy chain-like (MYH9) protein. The antibody, αMYH9, was further characterized and shown to bind circulating PGCs and embryonic gonadal PGCs (Hamburger Hamilton (H-H) stage 30, embryonic day 6.5–7). Whilst our primary aim was to determine the binding to PGCs, further investigation is required to determine potential binding to somatic cells. In conclusion, this study provides the characterization of a surface marker for chicken PGCs, with significant implications for advancements in avian genetic preservation, agriculture, and biotechnology.

## Simple Summary

This study focuses on improving the identification of chicken primordial germ cells (PGCs), which are vital for genetic transmission and biotechnological applications. Traditional markers like SSEA1 and CVH have limitations—SSEA1 lacks specificity, and CVH is intracellular. A monoclonal antibody was generated by injecting chicken PGCs into mice, producing one that specifically binds to PGCs and decreases with cell differentiation. Mass spectrometry identified its target as the MYH9 protein. The resulting αMYH9 antibody effectively labels PGCs at various developmental stages, offering a valuable tool for isolating viable PGCs and advancing avian genetics, agriculture, and biotechnology.

## 1. Introduction

With the global population continuously expanding, securing food production becomes increasingly critical, underscoring the importance of enhanced genetic engineering techniques in agricultural species. This has been validated through the development of transgenic crops, which have already demonstrated their value in agriculture by enhancing food quality and quantity over recent years.

The development of efficient methods for producing transgenic chickens is crucial due to their diverse applications in agriculture, biopharmaceutical industries, and academic research. In agriculture, the potential for disease-resistant transgenic birds holds immense promise for ensuring ongoing meat and egg production, as well as the potential for improving production traits [1,2]. Additionally, the emergence of zoonotic diseases like avian influenza highlights the urgent need to refine techniques for generating transgenic birds, as disease-resistant birds could potentially mitigate the risk of future avian influenza pandemics [2,3,4].

The accessibility of chicken embryos and their short incubation times also make them an excellent model for developmental biology research, offering valuable insights into crucial developmental processes [5,6,7,8]. Moreover, in the pharmaceutical sector, chickens serve as living bioreactors for generating biologically active proteins, such as tumor necrosis factor alpha antibody, which is used for treating conditions like rheumatoid arthritis [5,9,10]. Creating transgenic chickens capable of producing these proteins offers significant advantages to the pharmaceutical industry by enhancing economic viability.

Current methods of generating transgenic poultry primarily involve genetic manipulation of chicken primordial germ cells (PGCs), the precursors to gametes [11]. In mammalian embryonic development, the germ cells are located separately from the somatic cells and migrate through somatic tissues to the gonadal ridges. In avian embryonic development, however, the PGCs migrate via the vasculature system, allowing unique accessibility for collection and transplantation [12]. Therefore, PGC identification is critical to allow for efficient genome editing.

PGCs are identified using morphology, periodic acid–Schiff (PAS) staining, and various intracellular markers including chicken VASA homologue (CVH), chicken DAZL, chicken dead end homologue, and the surface marker SSEA1 [13,14,15]. Whilst SSEA1 is a valuable marker of PGCs, it has been demonstrated that its expression is not restricted to PGCs, and interestingly, not all PGCs express SSEA1 [16,17]. Using these methods of identification, it has been determined that there are between 200 and 400 PGCs found in germinal crescent (H-H stage 5, embryonic age = 19–22 h), following circulation in the vasculature system and into the genital ridge (H-H stage 19, embryonic age = 3–3.5 days).

There are many ways to manipulate the genomic DNA of PGCs, which includes the use of retroviruses, lentiviruses, stable transfections (using cultured PGCs), and site directed insertion of genes using transposons, all of which have a low transgenic efficiency. The limited proliferation efficiency and instability of culture systems present major obstacles [18], significantly hindering the use of PGCs in genetic breeding. To enhance the use of primary PGCs, novel surface markers are needed to improve PGC numbers by flow cytometry sorting and potentially aid in improving transgenic techniques. In this paper, we describe the generation of a monoclonal antibody that is specific to a marker on the surface of chicken primordial germ cells and the characterization of the marker.

## 2. Materials and Methods

### 2.1. Cell Lines and Cell Culture

In this study, we used two established PGC cell lines (611 and Fic1). These PGC cultures were established using peripheral blood PGCs harvested from day 2.5 (H-H stage 15) embryos. PGCs were cultured on gamma irradiated STO mouse embryonic fibroblast (ATCC CRL1503) based on a protocol previously described [19]. Briefly, once cultures were established, six-well cell culture plates were coated with gelatin 2 h prior to seeding irradiated (50 kGY) STO feeder cells with Knockout Dulbecco’s Modified Eagle Medium (KO DMEM) (Gibco Cat# 10829-018, Grand Island, NY, USA) containing 10% fetal bovine serum (FBS) (FisherBiotec Cat#S-FBS-AU-015, Wembley, Australia) and Glutamax (2 mM) (Gibco Cat# 35050-061) and then incubated at 37 °C 5% CO_2_ for 24 h. Cultured PGCs were then passaged 1 in 2 onto the feeder cells with KO DMEM (Gibco Cat# 10829-018) containing KO DMEM-BRL-3a conditioned media (20%) (ATCC CRL-1442), FBS (7.5%) (FisherBiotec Cat#S-FBS-AU-015), irradiated (50 kGY) chicken serum (2.5%) (Sigma Cat# C5405, Burlington, MA, USA), Glutamax (2 mM) (Gibco Cat# 35050-061), 2-Mercaptoethanol (130 µM) (Gibco Cat # 21985-023), glutamine synthetase expression medium (GSEM) supplement [1×] (Sigma Cat# G9785), mouse stem cell factor (mSCF) (6 ng/mL) (Peprotech Cat#AF250-03, Cranbury, NJ, USA), and human fibroblast growth factor (hFGF-2) (4 ng/mL) (Peprotech Cat# 100-18B) and cultured at 37 °C, 5% CO_2_, for 1 week.

### 2.2. Immunization and Hybridoma Production

All procedures described here were reviewed and approved by the Commonwealth Scientific and Industrial Organization, Australian Animal Health Laboratories Animal Ethics Committee (AEC#1401), and were performed in accordance with the Australian Code for the Care and Use of Animals for Scientific Research, 7th Edition, 2004. Cultured PGCs (5 × 10^6^ cells at a concentration of 1 × 10^8^ cells/mL) were washed 3 times in phosphate-buffered saline (PBS) injected subcutaneously (50 µL/site) at two sites into four 6-week-old male Balb/C mice in either Complete Freunds Adjuvant (F5881 Sigma) in a 1:1 (*v*/*v*) ratio for the first injection or with emulsified Incomplete Freund’s Adjuvant (F5881 Sigma) for the subsequent 3 immunizations in a 6-month time period, as per the manufacturer’s recommendations. Blood was collected using a 27-gauge needle from the lateral saphenous vein before initial injection (base line) and 7 days after each injection. Three days after the fourth immunization, a single mouse was chosen to be boosted intravenously with 5 × 10^6^ cells diluted in PBS (no adjuvant). The mouse was euthanized three days after the final boost, and the spleen cells were harvested and fused with Sp2 myeloma cells to generate hybridomas using the ClonaCell™-HY Hybridoma Kit (Stemcell Technologies Cat #03800, Vancouver, BC, Canada) liquid culture protocol. Briefly, Sp2/0-Ag14 (Sp2) (ATCC CRL-1581) were grown for a week in Medium A, passaging every second day, prior to the spleen being harvested. The spleen was disaggregated mechanically by pushing through a 70 µM sieve using a 3 mL syringe barrel insert. The sieve was washed with Medium B, and the spleen cells pipetted gently to form a single cell suspension. Sp2 cells were harvested, centrifuged at 300× *g* for 10 min, and resuspended in Medium B. Both the Sp2 cells and the splenocytes were washed 3 times in Medium B, centrifuging at 300× *g* for 10 min each time. Cells were counted using Trypan Blue stain, and volumes containing 2 × 10^7^ Sp2 cells and 1 × 10^8^ splenocytes were transferred to a 50 mL tube, mixed, and centrifuged at 400× *g* for 10 min. The supernatant was removed using a pipette so as not to disturb the cell pellet. Fusion was performed using the polyethylene glycol (PEG) method. ClonaCell™-HY PEG (1 mL) was added dropwise over 1 min to the intact pellet and then gently stirred for a further minute with the serological pipette used to dispense the PEG. Medium B (4 mL) was added to the PEG/Sp2/splenocyte mix and stirring continuously with the serological pipette for a further 4 min. Finally, 10 mL of Medium B was added, and the mixture was incubated at 37 °C for 15 min. After incubation, 30 mL of Medium A was added slowly and then centrifuged at 400× *g* for 7 min. The supernatant was removed with a pipette, so the cell pellet was not disturbed. The pellet was washed to make sure all PEG was removed using 40 mL of Medium A and centrifuged at 400× *g* for 7 min. The supernatant was removed using a pipette, and the cell pellet was resuspended in 10 mL of Medium C and transferred to a 75 cm^2^ flask containing 20 mL of Medium C incubated at 37 °C for 24 h. Cells were harvested from the flask, transferred to a 50 mL tube, and centrifuged at 400× *g* for 10 min. The supernatant was removed using a pipette, and the cells were resuspended in a final volume of 200 mL of ClonaCell™-HY Liquid Hat Selection Medium. Using a multichannel pipettor, 200 μL of cell suspension was added to each well of 10 × 96-well tissue culture plates and incubated at 37 °C and 5% CO_2_ for 10 to 14 days. Half of the medium was changed with selection media at day 7.

### 2.3. Hybridoma Selection

Supernatants were collected from hybridomas and incubated with PGCs overnight at 4 °C. Cells were washed once with PBS containing 2% (*v*/*v*) FCS and then incubated at 4 °C for 2 h with a FITC-conjugated anti-mouse secondary antibody (goat anti-mouse IgM/IgG/IgA (H + L) F(ab’)2-FITC Cat# AQ502F MerckMillipore, Darmstadt, Germany). The cells were analyzed three separate times using flow cytometry. and 12 positive clones were chosen to subclone using the limiting dilution method. Briefly, cells from positive clones were counted and diluted to give 5 cells/mL. Then, 200 µL of the diluted cells was transferred to give one cell per well. After 1–2 weeks incubation at 37 °C and 5% CO_2_, the wells were visually scanned for colonies. Subclones were rescreened using the same method to determine positivity. Of these clones, seven were isotyped using a mouse isotyping kit (Mouse Monoclonal Antibody Isotyping test kit (10 tests), AbD Serotec MMT1, Bio-Rad, Hercules, CA, USA).

### 2.4. Flow Cytometry and Cell Sorting

The following commercially available antibodies were used in this study: SSEA1 (480) mouse IgM, clone SC21702 (Santa Cruz Biotechnology, Dallas, TX, USA); goat anti-mouse IgG Alexa Fluor™ 488, clone A1101 (Thermo Fisher Scientific, San Diego, CA, USA); goat anti-rabbit IgG FITC, clone F0382 (Sigma-Aldrich Merck, Burlington, MA. USA); goat anti-mouse IgM Alexa Fluor™ 488, cloneA21042 (Invitrogen Thermo Fisher Scientific, Waltham, MA, USA); goat anti-rabbit IgG Rhodamine Red™-X, clone R6394 (Invitrogen Thermo Fisher Scientific); and goat anti-mouse IgM PE clone 1020-09 (Southern Biotech, Birmingham, AL, USA). An in-house developed CVH rabbit antiserum (a gift from Craig A. Smith, Monash University, Melbourne, Australia) was also used. Cells were incubated with the primary antibody for 1 h at room temperature (RT), washed (cells resuspended in PBS containing 2% FBS and centrifuged at 300× *g* for 3 min), and then incubated with appropriate secondary antibody for 1 h at RT and washed (cells resuspended in PBS containing 2% FBS and centrifuged at 300× *g* for 3 min). Finally, the cells were resuspended in 150 µL of PBS containing 2% FBS. The cells were then analyzed using an LSR II flow cytometer (Becton-Dickinson, Franklin Lakes, NJ, USA). Data were processed using BD FACSDiva software version 8.0.1 (Becton-Dickinson) or FlowLogic version 8.7 (Inivai Technologies, Birmingham, AL, USA). The cells determined to be positive for antibody binding were first-gated singlets using FSC-A versus FSC-H, selecting only events with an equal height and area. From these cells, a negative control was used to set parameters to determine fluorescent-positive cells. Cells were labeled as above and sorted using FACSAria II (Becton-Dickinson, Franklin Lakes, NJ, USA). The sorter conditions included using a 70 µM nozzle and cells sorted using a purity mask.

### 2.5. Quantitative Real-Time PCR (qRT-PCR)

RNA was extracted using the RNeasy Plus Mini Kit (Qiagen Cat#74134, Venlo, the Netherlands), and complimentary DNA (cDNA) was generated using SuperScript™ III First-Strand Synthesis SuperMix (Thermofisher Scientific Cat #18080400). A custom real-time primer/probe set and TaqMan™ Gene Expression Assay real-time PCR primer set were used to semi-quantitatively determine the gene expression of CVH (Custom Taqman gene expression assay Accession: NM_204708.1—probe sequence CACCACCTGTGCTTTGT FAM; forward primer sequence GGTTCCCCGGTTATCCAAGAG; reverse primer sequence TCCACAAACACCATGGTTCGTT), and glyceraldehyde-3-phosphate dehydrogenase (GAPDH) (Gg03346982_m1 Applied Biosystems, Foster City, CA, USA) was used as comparative housekeeping gene control. The relative quantitation of gene expression was determined using the StepOnePlus™ Real-Time PCR System (Thermo Fisher Scientific, San Diego, CA, USA), and the comparative threshold cycle (Ct) method was used to show changes in gene expression, according to manufacturer’s instructions (Applied Biosystems, Waltham, MA, USA). Relative gene expression was calculated using the mean values obtained in the formula ΔΔCt (Applied Biosystems) and as previously described [20].

### 2.6. Automated Immunohistochemistry Method Using PT Link Antigen Retrieval

Day 7 (H-H Stage 31) and day 14 (H-H Stage 40) chicken embryo gonads were fixed using 4% paraformaldehyde, paraffin embedded, and sectioned at 4 µm for Lillie–Mayer’s Hematoxylin and immunostaining (Agilent, Santa Clara, CA, USA). Prior to staining, antigen retrieval was performed using PT Link (DAKO, Glostrup, Denmark) and a high-pH target retrieval solution (Envision Flex Target Retrieval Solution High pH—DAKO). Immunostaining was achieved using the primary mouse monoclonal antibodies (4 µg/mL) and αCVH rabbit serum (1:500). Horseradish peroxidase (HRP)-linked α-mouse and α-rabbit secondary antibodies (Envision Flex (HRP)—DAKO) were bound and detected using AEC chromogen (DAKO). Sections were then stained with Lillie–Mayer’s Hematoxylin and mounted with an aqueous mountant (DAKO).

### 2.7. Immunocytochemistry

Cell preparation and staining. Gonadal PGCs were prepared by excising genital ridges from the mesonephros from embryonic age day 7 (H-H stage 31) or embryonic age day 14 (H-H stage 40) chicken embryos. Excess (non-gonadal) tissue was removed, and the gonads were placed in a 0.25% (*v*/*v*) trypsin–0.05% EDTA solution. Following gentle pipetting, the gonads were incubated at 37 °C for 7 min, gently pipetted, and incubated for a further 4 min at 37 °C. Medium containing 10% FCS was added 1:1 (*v*/*v*) to inactivate the trypsin/EDTA solution. Using a Cytospin 2 centrifuge (Shandon, Cork City, Ireland), disassociated gonadal PGCs were centrifuged onto microscope slides, fixed with 4% paraformaldehyde (PFA) (*w*/*v*), and permeabilized using 0.1% (*v*/*v*) TritonX100. The cells were blocked using PBS containing 5% BSA (*w*/*v*) and incubated for 1 h at RT. The cells were then incubated with primary antibody for 1 h at RT, washed with PBS, and incubated for a further 1 h at room temperature with the appropriate secondary antibody. Finally, cell nuclei were stained using DAPI (5 µg/mL) (Sigma, Burlington, MA, USA) for 5 min and the cells examined using fluorescence microscopy (Leica, Melbourne, VIC, Australia).

Cell injection and migration. Fertilized eggs from a transgenic chicken expressing the eGFP gene carried in a transposon [21] were incubated in an egg incubator at 37.6 °C and 60% humidity for 7 days. Genital ridges were carefully prepared as described above. Cells were labeled with αMYH9 and an α-mouse APC secondary and sorted as previously described. White leghorn chicken embryos at day 2.5 (H-H stage 15) were used as a host for the injection. A 1 cm diameter window was made on the blunt end above the air chamber to expose the embryos. PGCs were loaded into a glass micro needle and injected into the vasculature system of each embryo. The window was sealed by applying two layers of Parafilm (Bemis Company, Inc., Sheboygan Falls, WI, USA). The injected embryos were incubated for 6 days and then euthanized to isolate the gonads under a stereomicroscope. Images of the gonads were captured under an inverted fluorescent microscope (Leica, Melbourne, VIC, Australia).

All procedures described here were reviewed and approved by the Commonwealth Scientific and Industrial Organization, Australian Animal Health Laboratories Animal Ethics Committee (AEC#1690) and were performed in accordance with the Australian Code for the Care and Use of Animals for Scientific Research, 8th Edition, 2013.

### 2.8. Immunoaffinity Purification of Antigen

A lysate was prepared from the cultured PGCs using reagents from the Classic IP Kit (Cat#26146 Pierce Biotechnology Inc., Rockford, IL, USA) following the protocol provided. Briefly, cultured PGCs (4 × 107 cells, pellet weighing 350 mg) were lysed using ice-cold IP Lysis/Wash buffer (containing Halt™ Protease Inhibitor Cocktail [1×] 78429 Thermofisher Scientific) at 500 µL/50 mg cell pellet weight. To lyse, the cell/lysate mix was incubated on ice for 5 min and then centrifuged at 13,000× *g* for 10 min to remove cell debris. The total protein was quantified using Pierce BCA Protein Assay Kit (Cat # 23225) at 3.2 µg/µL. To preclear the 1.75 mL of lysate, 160 µL of Control Agarose Resin was placed in a column and centrifuged at 1000× *g* for 1 min, then washed with 200 μL of 0.1 M sodium phosphate and 0.15 M sodium chloride, pH 7.2, and centrifuged at 1000× *g* for 1 min. Lysate was added to the prepared Control Agarose Resin column and incubated at 4 °C for an hour with end-over-end mixing and centrifuged at 1000× *g* for 1 min. The flow through was collected. Purified αMYH9 mAb (10 µg) was incubated with 300 µL (1000 µg) of precleared cell lysate at 4 °C overnight. Protein A/G plus agarose (20 µL) was washed twice with 100 µL of cold IP Lysis/Wash buffer and centrifuged after each wash at 1000× *g* for 1 min. The lysate/antibody sample was added to the washed Protein A/G plus agarose and incubated for 2 h at RT with end-over-end mixing. The agarose was washed 3 times with 200 µL of cold IP Lysis/Wash buffer and centrifuged each time at 1000× *g* for 1 min. A final wash with 100 µL of 1× Conditioning Buffer was performed, following by centrifuging at 1000× *g* for 1 min. Finally, the immune complex was eluted using 50 µL of Elution Buffer (low pH) and incubated for 10 min at RT, centrifuged at 1000× *g* for 1 min, and the flow through retained.

### 2.9. Coomassie Staining and Western Blotting

Proteins were extracted using Lysis Buffer from the Classic Immunoprecipitation Kit (Cat#26146 Pierce Biotechnology Inc., Rockford, IL, USA) (containing Halt Protease and Phosphatase Inhibitor Cocktail (Cat#78429 Thermofisher Scientific)) and separated by SDS-PAGE (gradient gel 4–12%, run at 150 V for approximately 1 h). Gels were Coomassie stained using SimplyBlue™ Safe Stain (Life Technologies, Carlsbad, CA, USA) following the protocol provided. Briefly, the gels were washed once in water for 15 min at RT and then soaked for an hour in Simply Blue Safe stain. The gel was then destained for 3 h in water (changed every hour) and then imaged using Imagelab ChemiDocMP imager (Biorad, Hercules, CA, USA). For the Western blot, the resolved proteins were transferred using the Trans-Blot Turbo Transfer System, Mini (Cat # 1704150 Biorad) to PVDF membrane and blocked using 5% (*w*/*v*) skim milk powder (SMP) in phosphate-buffered saline with 0.05% Tween (PBST). Diluted αMYH9 mAb (1:500 in 5%SMP in PBST) was then added and incubated overnight at 4 °C, followed by secondary antibody, sheep anti-mouse HRP (1:5000 in 5%SMP in PBST) (Chemicon Australia, Boronia, Australia), and incubation for 2 h at 4 °C. Protein was detected using chemiluminescence (Clarity™ Western ECL Substrate, Cat#1705060 Bio-Rad, Hercules, CA, USA) and imaged using the ChemiDoc Imager (Biorad).

### 2.10. Identification of Antigens Using Mass Spectrophotometry

Protein identification. Coomassie-stained bands corresponding to the proteins of interest were excised and subjected to in-gel trypsin digestion, as previously described [22]. Gel bands were cut into 1 × 2 mm pieces, transferred to 0.5 mL Eppendorf tubes, and washed twice in 0.2 M NH_4_HCO_3_/50% acetonitrile. Protein in the gel pieces was then reduced with 10 mM dithiothreitol for 1 h in 0.2 M NH_4_HCO_3_, washed twice with 0.2 M NH_4_HCO_3_, and then alkylated with 50 mM iodoacetamide in 0.2 M NH_4_HCO_3_ for 20 min in the dark. Following two more washes in 0.2 M NH_4_HCO_3_ and a final wash in 0.2 M NH_4_HCO_3_/50% acetonitrile, the gel pieces were dried for 30 min in a SpeedVac concentrator. The gel pieces were then rehydrated twice with 10 µL of trypsin solution (modified sequencing grade, Roche) of 1 µg/20 µL in 50 mM NH_4_HCO_3_ for 10 min at 37 °C; then, 150 µL 50 mM NH_4_HCO_3_ was added, and digestion continued overnight at 37 °C. After overnight incubation, the sample was centrifuged at 12,000× *g* and the supernatant containing peptide fragments removed and kept aside. Residual peptides in the gel pieces were then extracted twice with 200 µL of 60% acetonitrile/1% trifluoroacetic acid (TFA) at 37 °C for 30 min, centrifuged, and the supernatants combined. Finally, the pooled supernatants were concentrated in a SpeedVac concentrator to approximately 50 µL for subsequent analysis by liquid chromatography–mass spectrometry or isolation by reverse-phase chromatography.

Tryptic fragments were subjected to LC-MS/MS analysis on a LTQ Orbitrap Elite mass spectrometer (Thermo Scientific) coupled to an Ultimate 3000 RSLC nanosystem (Dionex, Sunnyvale, CA, USA). The nanoLC system was equipped with an Acclaim Pepmap nano-trap column (Dionex) and an Acclaim Pepmap analytical column (Dionex) running on a 3–80% CH3CN-containing 0.1% formic acid gradient over 25 min. The mass spectrometer was operated in the data-dependent mode, whereby spectra were acquired first in positive mode followed by MS/MS fragmentation using collision-induced dissociation (CID). Ten of the most intense peptide ions with charge states ≥ 2 were isolated and fragmented using normalized collision energy of 35 and activation Q of 0.25. The Orbitrap MS data were analyzed using Proteome Discoverer (Thermo Scientific version 2.0) with the Mascot search engine against the Uniprot database. A false discovery rate threshold of 5% was applied.

### 2.11. Retinoic Acid Treatment and ML7 Treatment

For differentiation using retinoic acid (RA), cultured PGCs were grown, as above, in 6 wells of a 6-well plate (2 wells with retinoic acid (diluent ethanol) treatment, 2 with wells diluent alone, and 2 wells untreated) seeded with approximately 1 × 10^5^ cells per well, with and without 1 µmol/L retinoic acid (R2625 Sigma-Aldrich), for 72 h [23]. To interrupt MYH9 expression, cultured PGCs were grown, as above, in 6 wells of a 6-well plate (2 wells witih 1-(5-Iodonaphthalene-1-sulfonyl)-1H-hexahydro-1,4-diazepine (ML-7) (diluent ethanol) treatment, 2 wells with diluent alone, and 2 wells untreated) seeded with approximately 1 × 10^5^ cells per well, with and without 20 µM ML-7 (I2764 Sigma-Aldrich), for 6 days.

### 2.12. Statistical Analysis

Statistical differences of gene expression between different cell populations were determined by One-way ANOVA Tukey’s Multiple-Comparisons test.

## 3. Results

### 3.1. Generation and Selection of Mouse Monoclonal Antibodies Against Chicken Primordial Germ Cells

**Figure 1:**
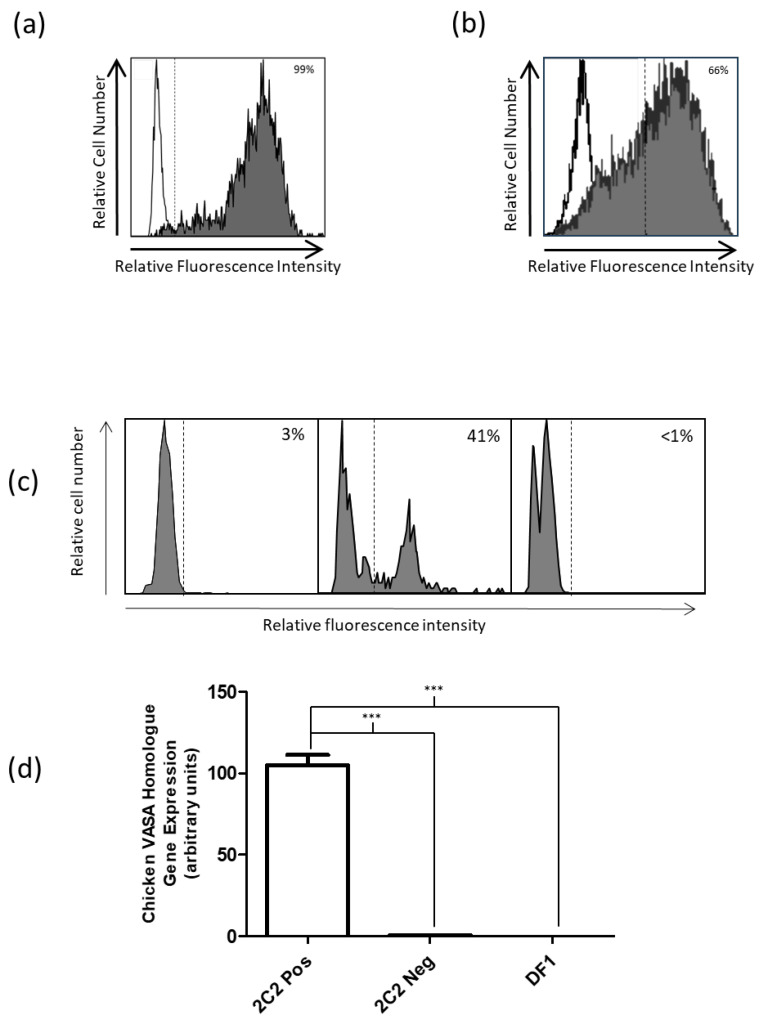
The 2C2 clone binds the majority of chicken PGCs, SSEA1 shows comparable binding, and 2C2-positive cells express high levels of CVH. (**a**) The histogram shows a representative plot of flow cytometric analysis of cultured chicken primordial germ cells (PGCs) bound to mouse monoclonal antibody (mAb) generated against a surface marker on chicken PGCs. (**b**) The histogram shows a representative plot of flow cytometric analysis of cultured PGCs bound to stage-specific embryonic antigen-1 (SSEA1) surface marker. (**c**) Flow cytometric labeling of the surface on peripheral blood PGCs and sorted 2C2-positive and 2C2-negative cells. The histogram shows a representative plot of 2C2-positive cells enriched from the peripheral blood of a 2.5-day- (Stage 17 Hamburger–Hamilton (H&H)) old embryo using 2C2 antibody. (**d**) Real-time PCR showing chicken VASA homologue (CVH) gene expression, with 195 increased fold change for 2C2-positive sorted compared to 2C2-negative sorted and 3 × 10^5^ increased fold change when compared to chicken fibroblast (DF1) cells (One-way ANOVA Tukey’s Multiple-Comparisons test *p* < 0.0001 (***)).

Hybridomas were generated (in total of 960 wells) and screened with cultured PGCs using flow cytometry. Samples were called positive if >95% of the PGC population had bound antibody. Of the 960 wells, 48 were selected to continue culturing. After screening a further 4 times, 12 clones were chosen to subclone, and of the subclones, 7 were chosen for continued analysis. These clones were isotyped, which showed that four clones were identified as IgG1 (2C2, 7E2, 2E9, and 3G5) and three as IgM (1B12, 6A3, and 1D3). After preliminary characterization of the IgG1 monoclonal antibodies, the clone 2C2 was chosen to investigate further. Following purification of the 2C2 clone, we reanalyzed its binding to cultured chicken PGCs by flow cytometry and demonstrated binding at 99% (Figure 1a). This was compared to stage-specific embryonic antigen-1 (SSEA-1), which showed 66% binding to cultured PGCs (Figure 1b).

**Figure 2:**
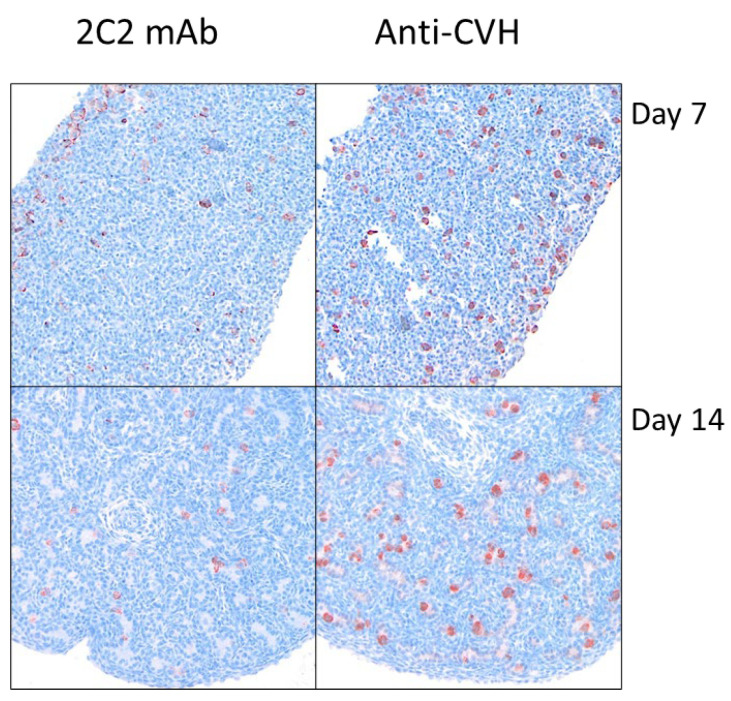
2C2 mAb staining reduces during gonadal development, conversely to CVH expression. Paraffin embedded gonads from day 7 (Stage 31 Hamburger–Hamilton (H&H)) gonads (10×) and day 14 (Stage 40 H&H) gonads (20×) stained using 2C2 monoclonal antibody (mAb) and anti-chicken VASA homologue (αCVH) mAb (secondary Ab—Envision Flex Horseradish Peroxidase (HRP) and 3-Amino-9-Ethylcarbazole (AEC) chromogen (red)) with Lillie–Mayer’s Hematoxylin counterstain (blue).

To determine if the surface marker that 2C2 detects was not a cell culture anomaly and is expressed on primary PGCs, peripheral blood PGCs were sorted using the 2C2 clone, and CVH gene expression was analyzed using quantitative real-time PCR (qRT-PCR). Following cell sorting, only 41% of cells were positive for 2C2, potentially suggesting that the sorting process impacted the antigen expression (Figure 1c). Total RNA was extracted from the positive and negative sorted populations from whole peripheral blood from 2.5-day-old embryos. The CVH gene expression in the 2C2 positively sorted population had a significantly higher expression when compared with the antibody-negative population (195 increased fold change, *p* < 0.0001) and DF1 (3 × 10^5^ increased fold change, *p* < 0.0001)) indicating the positive population containing PGCs (Figure 1c). The immunohistochemical staining patterns of 2C2 and CVH were compared using day 7 and day 14 gonads stained with each antibody. We observed similar staining patterns in day 7 gonads; however, day 14 gonads contained CVH-positive cells, but their 2C2 expression was reduced (Figure 2).

**Figure 3:**
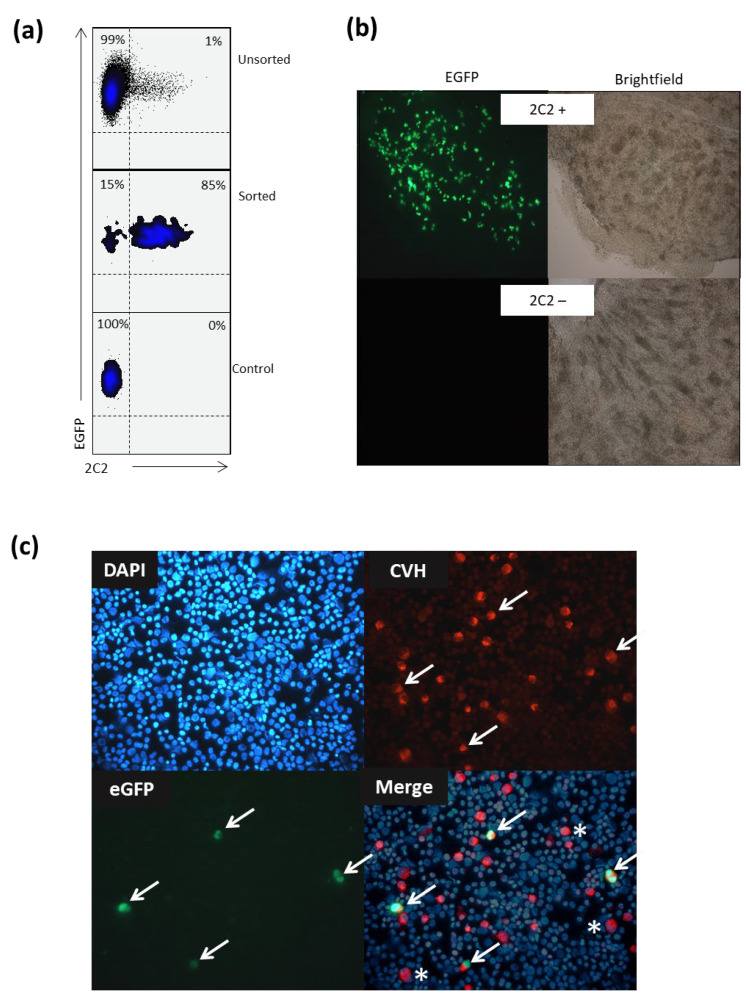
2C2-positive cells migrate to the genital ridge. (**a**) Flow cytometry histograms represent unsorted, 2C2 sorted, and control enhanced green fluorescent protein (eGFP) primordial germ cells (PGCs) stained with the 2C2 mAb. (**b**) Representative brightfield and fluorescent images of whole-mounted gonads of recipient non-eGFP chickens injected with eGFP gonadal cells positively sorted using 2C2 monoclonal antibody (mAb) (10×). (**c**) Cytospot from dissociated gonads with migrated 2C2 mAb sorted cells from an eGFP donor chicken to determine if the chicken VASA homologue (CVH) is expressed in the migrated eGFP gonadal PGCs and endogenous PGCs. (i) 4′,6-diamidino-2-phenylindole (DAPI) stain (blue), (ii) eGFP gonadal PGCs (green), (iii) CVH Rhodamine Red (red), and (iv) DAPI/eGFP/Rhodamine Red merged. Arrows indicate injected eGFP PGCs, and [*] indicates endogenous PGCs (40×).

PGCs circulate in the peripheral blood and then migrate to the genital ridge. To ascertain that 2C2-positive cells can home to the genital ridge, 2C2-positive and -negative gonadal PGCs from EGFP-expressing birds were sorted and reinjected into the peripheral blood of non-EGFP birds (Figure 3a). The gonads from day 7 embryos were removed and whole mounted onto slides. The gonads from all six birds injected with 2C2-negative cells showed no EGFP-positive cells, whereas the gonads from all six birds injected with 2C2-positive cells displayed EGFP-positive cells (Figure 3b). To further confirm that the 2C2-positive cells that migrated to the gonads were PGCs, the gonads were trypsinized, cytocentrifuged, and stained for CVH (Rhodamine Red). The merged image shows that all EGFP-positive cells in the gonads were CVH positive and that there were additional CVH-positive cells that were not EGFP positive, likely endogenous PGCs (Figure 3c).

### 3.2. Identification of the 2C2 Antigen

**Figure 4:**
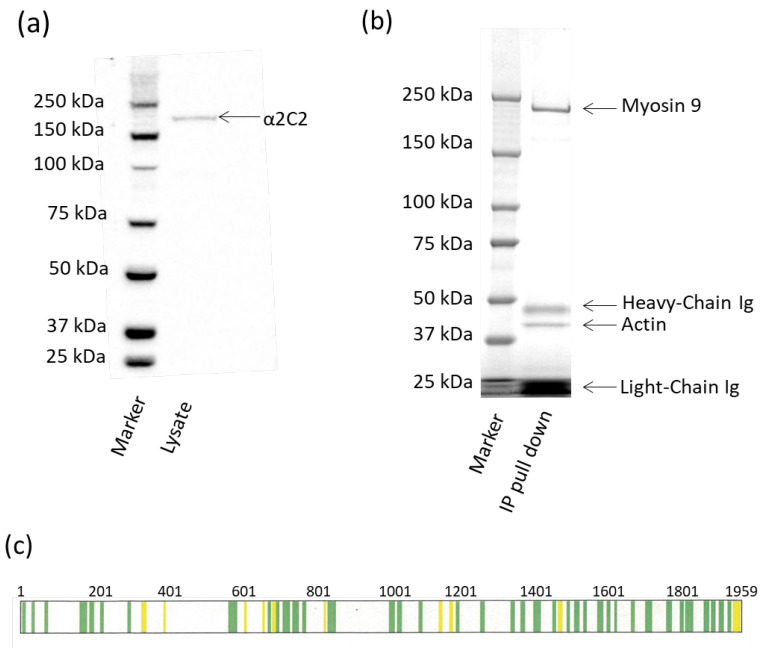
The 2C2 mAb detects cell surface myosin heavy chain 9 (MYH9). (**a**) Western blot analysis of primordial (PGC) cell lysate using MYH9 monoclonal antibody (mAb) and Western C Plus marker detects a 226 kDa band. (**b**) Coomassie-stained PAGE gel shows immunoprecipitate (IP) pull down from PGC lysate. (**c**) Schematic representation of amino acids identified by the 2C2 mAb shows peptides of chicken myosin-9 protein sequence (1959 amino acids, Accn # P14105) identified in sequencing, with a false discovery rate (FDR) of 1% (green) and 5% (yellow).

To determine the antigen that PGC mAb 2C2 binds to, a Western blot was performed using cell lysates from cultured PGCs. Figure 4a shows that the 2C2 mAb binds to a large protein (approximately 226 kD). To determine the identity of the protein that the 2C2 mAb bound to, immunoprecipitation was performed, and subsequent SDS-PAGE gel analysis was performed (Figure 4b). The Western blot showed binding to a single protein, and the SDS-PAGE analysis showed four distinct bands, corresponding ~226 kDa, ~50 kDa, ~45 kDa, and ~25 kDa. Eluates from the immunoprecipitation were analyzed on a 3-8% TrisAcetate PAGE gel, and bands were cut out and subjected to mass spectrometry (Figure 4c). It was found that the 226 kDa antigen to which 2C2 mAb bound was chicken myosin heavy chain 9, non-muscle (IIa) (MYH9). Protein sequencing coverage of the band identified by the MYH9 antibody shows peptides covering 26.24% of chicken myosin-9 protein sequence (1959 amino acids, Accession P14105) with a false discovery rate (FDR) of 1% (representative data only shown in green). Three other bands were identified in the immunoprecipitate as mouse monoclonal antibody heavy chain (~50 kDa), chicken actin (~45 kDa), and mouse monoclonal antibody light chain (~25 kDa).

**Figure 5:**
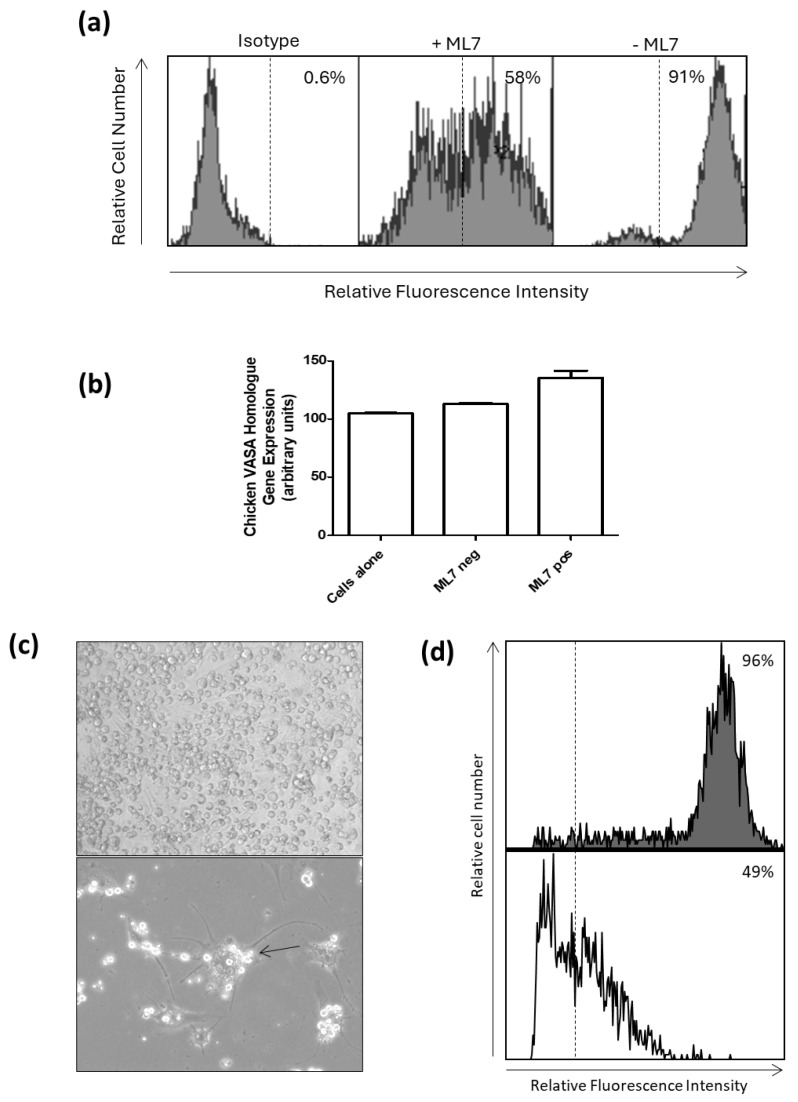
Myosin is downregulated on the surface of PGCs following ML7 treatment, and PGC differentiation leads to reduced MYH9 expression. (**a**) The histograms show the proportion of cells expressing either myosin heavy chain 9 (MYH9) in the presence or absence of the ML7 (Hexahydro-1-[(5-iodo-1-naphthalenyl)sulfonyl]-1H-1,4-diazepine hydrochloride). Markers are set against the relevant isotype control. (**b**) The bar graph shows qPCR data of the chicken VASA homologue (CVH) gene expression. (**c**) Brightfield images of cultured chicken primordial germ cells (PGCs) untreated and treated with retinoic acid (RA) for 72 h (40×). The arrow indicates morphologically neuronal-like cells. (**d**) Flow cytometric analysis of RA-treated and-untreated cultured chicken primordial germ cells (PGCs) labeled with the MYH9 mAb. Antibody-positive cells are to the right of the dashed isotype line.

To further confirm that the antigen was MYH9, a known myosin inhibitor, ML7, was used to treat PGCs, and the expression was evaluated using binding of the 2C2 mAb by flow cytometry. Binding with the 2C2 mAb was reduced from 91% in untreated PGCs to 58% in ML7-treated PGCs (Figure 5a). To show that this was not due to PGC differentiation, real-time PCR of CVH was used and confirmed that the PGCs had maintained a germ cell state despite treatment with ML7 (Figure 5a). From this, we concluded the antigen to be MYH9 and will now refer to the 2C2 clone as αMYH9.

To further determine that the antigen of αMYH9 was a marker of undifferentiated stem cells (i.e., PGCs) and not ubiquitously expressed, we treated PGCs with retinoic acid to induce differentiation (Figure 5c). The treated and untreated PGCs were analyzed by flow cytometry, and it was found that the percentage of positive cells was reduced from 96% (untreated) to 49% (treated), suggesting that upon differentiation, the cells appeared to have reduced expression of the surface marker detected by the PGC mAb αMYH9 (Figure 5d).

## 4. Discussion

To date, PGCs are identified by their large size, large round nuclei, and refractive lipids in their cytoplasm, along with the periodic acid–Schiff glycogen stain. There are also many nuclear/cytoplasmic markers that are specific to PGCs, which include the most specific germline cell marker, the RNA-binding factor gene called CVH. The surface marker SSEA1, which also stains chicken embryonic stem cells, is not exclusive PGCs and does not stain all PGCs [16,17]. This leaves a gap in specific PGC identification using surface markers. Surface markers are important in the identification and purification of cells, as they can remain viable for further culture and analysis. In the case of generating transgenic chickens, the ability to sort viable PGCs for genetic manipulation could potentially improve this process.

The MYH9 monoclonal antibody was generated by injecting whole cultured PGCs into mice, and the hybridoma supernatant was screened using cultured PGCs. Despite using cultured PGCs to generate the MYH9 antibody, we have demonstrated that it also binds to both gonadal and peripheral blood PGCs. However, when staining day 14 gonads (H-H stage 40), there appears to be some gonads where staining of the PGCs is reduced compared to CVH. PGCs are often isolated to sections of the gonadal tissue, that is, not dispersed uniformly across gonadal tissue, which may explain some histological sections with reduced PGC staining [24]. This may also be due to the differentiation of the PGCs to gametes. Gonadal germ cells differentiate to oogonia on day 8 and spermatogonia at day 13, which may explain the loss of antigen at HH stage 35–39 [25,26], unlike CVH expression, which is maintained in gametes [27]. This warrants further investigation, where the gender of the embryo from which the gonad is harvested is known.

It was determined by immunoprecipitation that the antigen to which MYH9 mAb bound was myosin, heavy chain 9, non-muscle MYH9 (myosin II). This was quite an interesting find, as it appears to be rare for myosin to be found on the cell surface. It has been reported that myosin is involved with the cell membrane [28] and that, potentially, a portion may be located on the surface of the cell [29]. However, this was reported in cultured cells and, therefore, could potentially be a cell culture anomaly. As we used cultured PGCs as our immunogen in generating the antibody, it was important to show that the antibody did in fact bind to primary gonadal PGCs and peripheral blood PGCs. Myosin II has also been suggested to act as a receptor for herpes simplex virus, where it interacts with glycoprotein B [30,31]. It is suggested that the tail portion of the myosin is embedded in the membrane, and the head portion is exposed in the cytoplasm to interact with actin. It should also be noted that the difference between myosin I and II is that myosin I lacks the coiled-coil tail [32], which is suggested to interact with the membrane, which lends further evidence that αMYH9 is binding to myosin II where the tail is exposed on the surface of the cell. Myosin II is a protein involved in various cellular processes like cell migration, adhesion, and cytokinesis. It is an actin-based motor protein, meaning that it interacts with actin filaments to generate force and movement within cells. Myosin II is widely expressed, and its dysregulation is implicated in several diseases, including cancer. Cell surface expression of myosin II may be limited to a small number of cell types and physiological states.

Potentially myosin II could be present in PGCs due to the need to migrate through the blood vessel endothelium to the gonadal ridges. It has been shown that the migration of chicken PGCs uses a similar chemokine homing process as that used in metastasis [33]. Interestingly, an upregulation of myosin II led to decreased survival rates in lung adenocarcinoma patients [34], and this was also observed in aggressive breast cancer subtypes [35,36], where poor prognosis is linked with metastasis. In metastatic breast cancer, myosin II was predominantly found at the invasive edges in cancer tissue and appeared to be required in breast cancer cell lines for invasion and dissemination, which could be a similar process to peripheral blood PGCs migrating through the endothelial tissue.

## 5. Conclusions

In conclusion, we have generated an antibody that binds to the surface of the chicken primordial germ cell that may have the ability to enhance chicken germline preservation, selective breeding, and the generation of genetically modified birds. Further characterization of this surface marker could shed light on its role in PGC biology, particularly regarding its expression patterns during gametogenesis. Additionally, employing this antibody to isolate PGCs from other avian species warrants exploration, especially given that reagents are limited.

## Data Availability

The original contributions presented in this study are included in the article. Further inquiries can be directed to the corresponding author.
